# Failure probability analysis of high fill levee considering multiple uncertainties and correlated failure modes

**DOI:** 10.1038/s41598-024-59762-5

**Published:** 2024-04-24

**Authors:** Ruirui Sun, Yimingjiang Reheman, Xiaoling Wang, Kaixuan Fei, Jinjun Zhou, Ding Jiao

**Affiliations:** 1 Faculty of Architecture, Civil and Transportation Engineering, Beijing University of Technology, 100 Pingleyuan, Chaoyang District, Beijing, 100124 China; 2 State Key Laboratory of Hydraulic Engineering Simulation and Safety, Tianjin University, Tianjin, 300072 China

**Keywords:** Long-distance water transfer project, Open channel levee, Failure probability model, Probabilistic and non-probabilistic approaches, Copula method, Natural and epistemic uncertainty, Civil engineering, Natural hazards, Hydrology

## Abstract

Such complex causative factors in current failure probability models are represented by simply random uncertainty and completely independent or correlation of failure modes, which can often limit the model utility. In this study, we developed a methodology to construct failure probability models for high fill levees, incorporating the identification of uncertainties and an analysis of failure modes. Based on quantification of stochastic-grey-fuzzy uncertainties, probability analysis involved with overtopping, instability and seepage failure modes was implemented combined with probability and non-probability methods. Given that the interaction among failure modes typically exhibits nonlinear behavior, rather than linear correlation or complete independence, a simple methodology for the binary Copula function was established and implemented in MATLAB. This methodology was applied to the high fill segments of a long-distance water transfer project characterized by high population density. It shows that the failure probability of a single failure mode is overestimated when uncertainties are not considered, because of the randomness and fuzziness of some parameters and the greyness of information. Meanwhile, it is found that the magnitude of failure probability related to levee breach is overestimated without respect to failure modes correlation, especially when the probabilities of seepage and instability are both significant and closely aligned.

## Introduction

Long-distance water transfer projects are required to alleviate the contradiction between supply and demand for water resources in different regions, realize rational water resource management^1^. Open channels, as critical components of these projects, particularly in areas with high fill levees, are subject to complex causative factors that introduce uncertainties and nonlinear correlations, significantly impacting the probability of failure. Consequently, breaches in channel levees pose substantial risks to public security, especially in densely populated urban areas.

Several factors, including soil erosion and catastrophic floods, predispose levees to breaches. Risk analysis has emerged as an essential tool for enhancing the safety and management of open channel levees. The applicability of risk analysis in the water system was initially demonstrated by Yen and Ang^2^, with subsequent widespread adoption in hydraulic engineering^3–5^. Early studies concentrated on water quality and operational risks of water transfer projects, employing various assessment methods such as the drivers-pressures-state-impact-response model, fuzzy comprehensive evaluation, and coordinated development degree model^6^. The improved failure mode and effect analysis method based on fuzzy inference system was utilized to the risk assessment of the Middle Route of the South-North Water Transfer Project^7^.

Currently, the safety assessment of levees increasingly relies on failure probability models. Lendering et al.^8^ proposed an approach to quantify the failure probability of flood control facilities, enhancing canal levees' reliability analysis. Similarly, Hathout et al.^9^ developed a model based on expert judgement to evaluate the failure probability of river levees. It is generally recognized that risk is intricately linked to uncertainty, risk assessments typically characterize inherent stochastic uncertainties through statistical probabilities.

In the risk assessment, the inherently stochastic uncertainty from the system itself was usually characterized with statistical probability, that is, the failure probability^10^. A unified framework incorporating both probabilistic and non-probabilistic methods has been established for representing uncertainties, embracing approaches like sampling-based methods, asymptotic reliability analysis, interval analysis, and fuzzy set theory^11,12^. Given the diversity of problems encountered in uncertainty analysis, no single method suffices for all scenarios. Hence, integrating probabilistic and non-probabilistic methods offers a more comprehensive strategy for flood risk management in open channels with high fill areas.

Open channel safety and levee breach risk management depend on quantitative analyses fraught with significant uncertainties. Recently, a considerable interest is focused on uncertainty analysis in hydrology, water quality and water resource fields^13,14^. From the risk probability perspective, some researches were performed on single uncertainty analysis of operation risk for reservoirs with respect to hydraulic engineering. The stochastic uncertainty was primarily considered in risk assessment of water transfer projects. As the most widely used method to describe uncertainty, mathematical statistics method is developed from the initial direct integration method, Monte Carlo method to the first-order reliability method (FORM) and second-order reliability method, mean FORM, and JC method, etc.^15^.

While stochastic uncertainty has been a primary consideration, the complexities of water conservancy projects also introduce fuzzy and grey uncertainties. The operation of water conservancy engineering systems involves a variety of uncertainties, making the analysis and description of the relationship between uncertainty and risk challenging. Recently, increased attention has been given to compound uncertainty, which arises from the interaction of two or more uncertainties. The main sources of uncertainty are analyzed and determined as uncertain dam breach and flood routing processes^16^. Based on fuzzy set theory, a new dam failure probability model was introduced combined with event tree analysis^17^. Additionally, Oliver et al.^18^ established an efficient modelling framework to perform probabilistic description of dike-protected river system taking morphological variability and stochastic uncertainty into account. Moreover, a probabilistic risk assessment method addressing Grey-Stochastic-Fuzzy uncertainty was applied to a roller-compacted concrete dam, considering both the random and grey attributes of parameters and the fuzziness of failure criteria^19^, with fuzzy failure criteria examined through enhanced LHS sampling methods and grey uncertainty quantified via Bootstrap Grey Estimation theory. Recently, an important advance in assessing failure probability under epistemic and aleatory uncertainties is a series of works on extended polynomial chaos expansions^20,21^. By focusing on the sensitivities to both aleatory and epistemic uncertainties, this approach offers valuable insights into the factors most influencing system performance and failure probabilities^22,23^. The aforementioned researches underscore the growing focus on addressing both natural and epistemic uncertainties, beyond merely the inherent stochastic uncertainty, in the analysis of dam and river levee failure probabilities. However, identifying and quantifying the failure probability of high fill levees in long-distance water transfer projects remains challenging due to the complex causative factors related to hydrological and geotechnical variability, as well as operation conditions. In addition, the simplistic representation of failure modes as either completely independent or correlated in detailed failure probability models can restrict their effectiveness. Therefore, analyzing the correlation among failure modes is crucial for deriving an accurate estimate of failure probability.

Correlation analysis of failure modes is vital for developing failure probability models for channel levees in long-distance water transfer projects. There has been a notable increase in studies focusing on failure probability models in dam and levee engineering that consider specific failure modes during the operational phase. Failure mode correlation analysis has recently been applied to the study of failure probability in structural engineering. For example, as for bridge structure reliability analysis, Liu and Fan^24^ presented the mixed copula models for time-independent reliability analysis of series, parallel, series–parallel, and parallel-series systems for two-component systems and multi-component systems with multiple failure modes. Gong and Frangopol^25^ applied Copula functions to describe the spatial correlation of corrosion growth associated with different girders to investigate the effect of spatial dependence of general corrosion on the reliability of steel girder systems under traffic loads. Furthermore, the correlation between slope failure modes has been accurately depicted using Pearson correlation coefficients, and the upper and lower limits were narrow to effectively reflect the change of system failure probability, and correlation between soil cohesive strength and friction angle was represented though Copulas^26^. Despite these advancements, understanding the nonlinear correlation among failure modes in high fill parts of open channel levees remains a significant challenge in failure probability analysis.

To sum up, the long-distance water transfer project levee's failure probability model was established, taking into account the multiple uncertainty, to obtain the failure probability of the channel levee. And the uncertainty was quantified by combining both probabilistic and non-probabilistic methods. Furthermore, the Copula function was used to calculate the integrated failure probability of the levee by considering the correlation among different failure modes.

## Methodology

**Figure 1:**
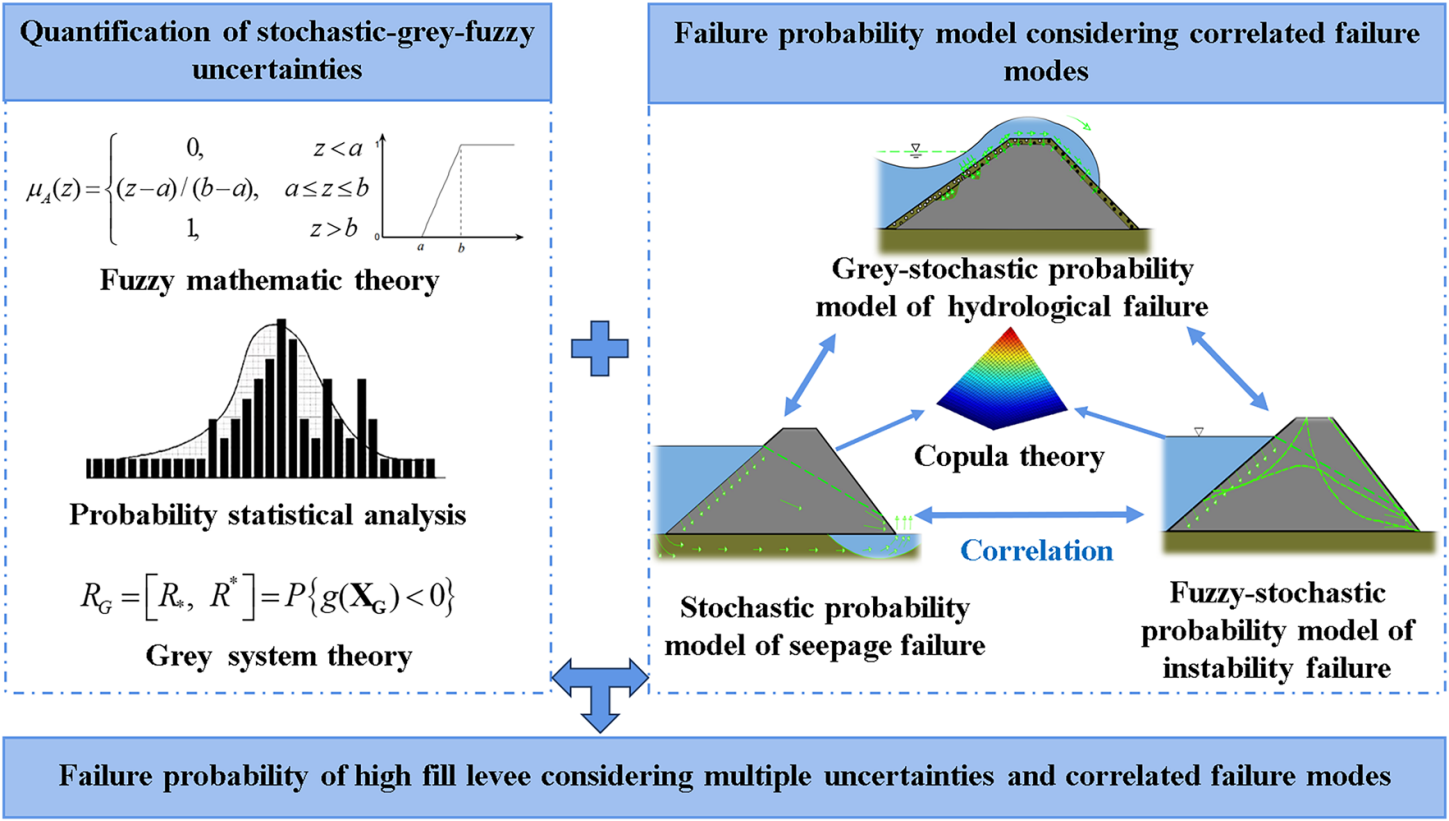
Failure probability analysis model for high fill levees.

In this paper, failure modes of channel levee with complex causative factors involved multiple uncertainties and nonlinear correlation was studied utilizing probability and non-probability methods, enabling a correlated analysis of failure probability in high fill parts. Initially, the identification of uncertainties related to these complex causative factors was carried out, distinguishing between natural and epistemic uncertainties. The development of a failure probability model incorporated the three primary failure modes: hydrological, seepage, and landslide instability, employing fuzzy mathematics and grey theory. Notably, the grey uncertainty associated with hydrological risk was quantified using the Dempster-Shafer evidence theory, while a rising half trapezoidal distribution of fuzzy membership degree was utilized to address the fuzzy uncertainty in instability failure risk. Additionally, a simplified methodology employing the binary Copula function was devised and implemented in MATLAB using the Gaussian Copula method, acknowledging the nonlinear interaction of failure modes, which contrasts with the linear correlation or complete independence typically assumed in traditional reliability analyses. As a case study, a high fill section of a long-distance water transfer project situated in a densely populated area was examined. The development of the failure probability analysis model for high fill levees is illustrated in Fig. 1.

### Uncertainty identification related to complex causative factors of high fill levee

Actually, during the operation of the long-distance water transfer project, the integrity of the channel is inevitably compromised by a multitude of uncertain factors, attributable to extensive water conveyance lines and complex engineering designs. Breaches in levees can lead to severe consequences. Therefore, to mitigate losses and manage risks, it is crucial to conduct a comprehensive analysis of the various uncertainties involved in the risk assessment of high fill channel levee breaches. Although uncertainties emanate from numerous sources, it is essential to distinguish between two primary categories: natural uncertainty (includes stochastic and fuzzy uncertainty) and epistemic uncertainty (includes grey and unascertained uncertainty)^27,28^. Grey uncertainty arises from incomplete knowledge about the system, which, being an integrated entity with specific functions comprised of interrelated and interactive elements, cannot be fully understood due to this knowledge gap. Uncertainties often coexist and interact, complicating the identification and significance assessment of each type^29^. Therefore, considering only one type of uncertainty in isolation is inadequate for addressing the multifaceted nature of uncertainty in risk assessments. To tackle the compounded uncertainty stemming from the interaction of multiple uncertainty types, a thorough identification and analysis of these uncertainties are imperative.

Conducting an uncertainty analysis for every potential variable would render risk assessments prohibitively time-consuming and inefficient. Thus, simplification and assumption are necessary to streamline the process. In practical engineering scenarios, uncertainties are frequently obscured by complexity. Specifically, in the operation of open channel high fill levee projects, uncertainties arise from variations in load effects, material strength resistance, and factors related to design and construction. Building on existing research^13,30–32^ and considering the unique aspects of high fill channel projects, uncertainties during the operational phase of open channels have been categorized as presented in Table 1.

**Table 1:** Uncertainties in the high fill levees.

Types of uncertainty	Including phenomena	Closely related factors	Uncertainty categorization
Hydrological uncertainty	Distribution of rainstorm and annual rainfall, etc.	Storm flood	Stochastic uncertainty and grey uncertainty
Earthquake factors uncertainty	Earthquake strength, intensity, source, action	Seismic factors	Grey uncertainty and unascertained uncertainty
Hydraulic uncertainty	Physical quantities that possess a property of uncertainty when calculating the hydraulic load	Technology eigenvalues and model simplification	Fuzzy uncertainty and unascertained uncertainty
Geotechnical uncertainty	Geological structure, piping, seepage, settlement and slope stability	Technological factors	Stochastic uncertainty and fuzzy uncertainty
Structure and construction factors uncertainty	Incorrect design, construction materials strength, and the relatively poor construction quality	Human factors	Fuzzy uncertainty and unascertained uncertainty
Operations management factors uncertainty	The degree of engineering maintenance, improper operation and human negligence in the process of management	Human factors	Fuzzy uncertainty and unascertained uncertainty

This study categorizes the failure types that can lead to breaches in filled channel levees as follows: hydrological failures (encompassing flood overflow and overtopping), seepage failures, and instability failures. The hydrological failures are attributed to the stochastic uncertainty of natural uncertainty and the grey uncertainty and unascertained uncertainty of epistemic uncertainty. The uncertainty that caused hydrological failure includes the stochastic uncertainty of natural uncertainty and the grey uncertainty and unascertained uncertainty of epistemic uncertainty. Similarly, seepage and instability failures stem from the fuzzy uncertainty of natural uncertainty and the unascertained uncertainty of epistemic uncertainty. Consequently, this paper explores the integrated failure probability of channel levees by analyzing these uncertainties and considering the influence of multiple factors.

### Failure probability model of the high fill levee based on probabilistic and non-probabilistic approaches

**Figure 2:**
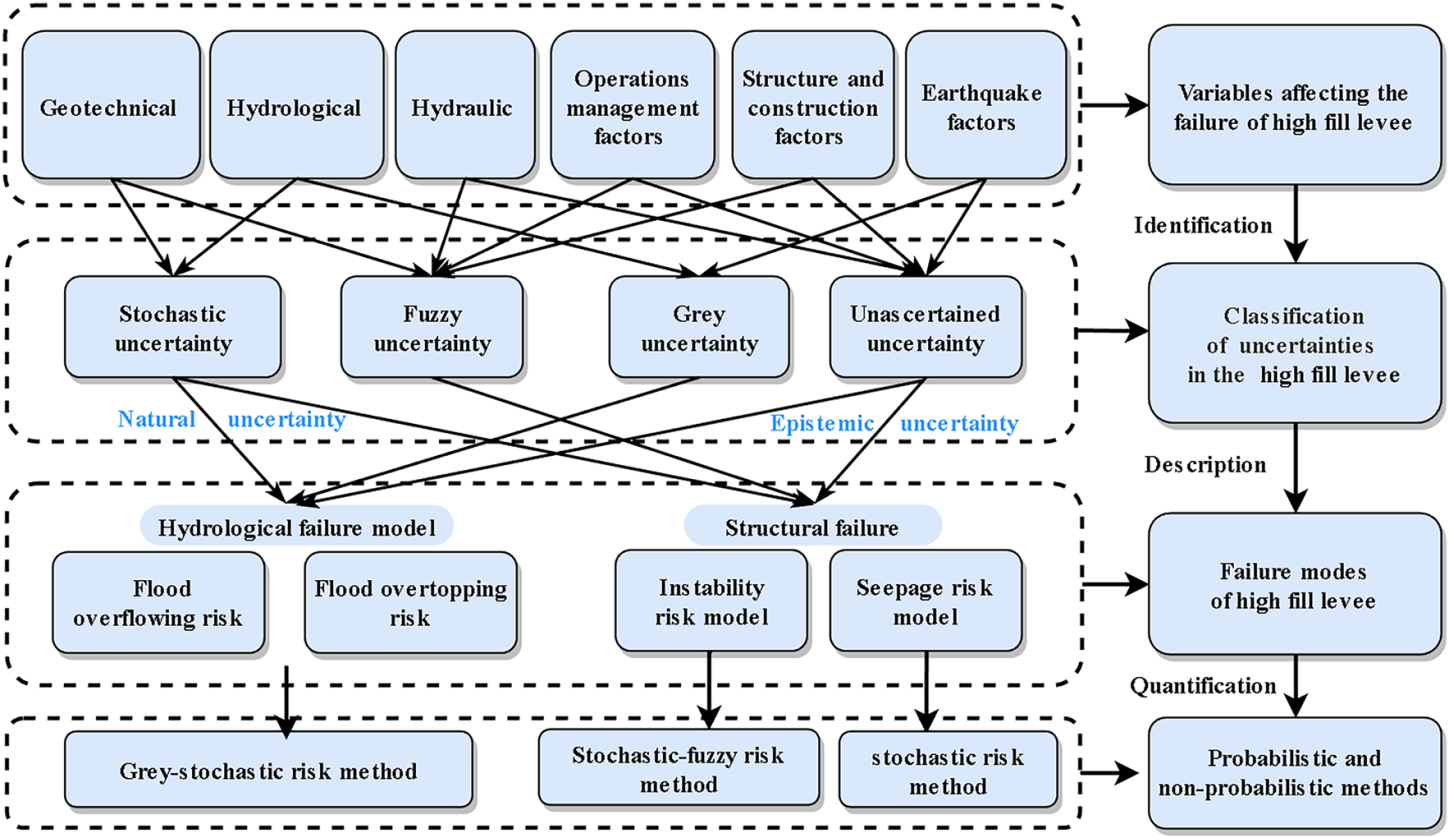
Identification and quantification of uncertainties in high fill levees.

The subjective uncertainty associated with levee breaches can be quantified using statistical theory, fuzzy mathematics, and grey system theory, as illustrated in Fig. 2. Statistical theory quantifies the risk of subjective uncertainty as a probability distribution, assuming statistical significance. Fuzzy mathematics represents the concept of failure probability as fuzzy probabilities^33^, while grey system theory focuses on describing and quantifying grey uncertainty in failure modes through a stochastic probability approach. The probability of levee breach caused by hydrological failure can be described by the grey-stochastic failure probability. In contrast, the probability of a breach due to instability failure is determined using stochastic-fuzzy failure probability, and the probability related to seepage failure is calculated with stochastic failure probability.

#### Hydrological failure probability model considering grey-stochastic uncertainty

**Figure 3:**
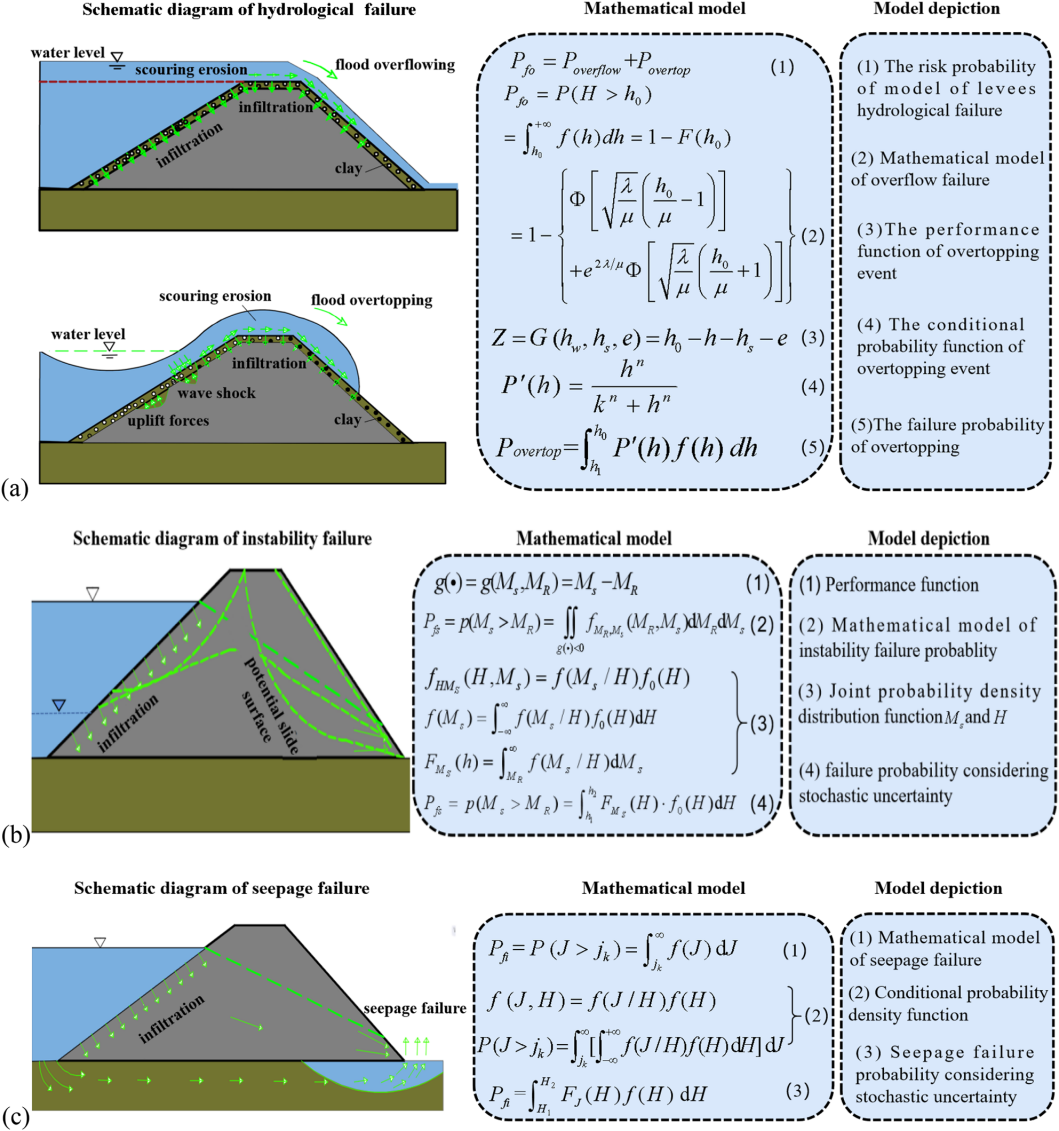
Mathematical model of levee failure risk analysis. (**a**) hydrological failure (**b**) instability failure (**c**) seepage failure.

The risk probability model for hydrological failure of levee is presented in Fig. 3a. The grey uncertainty risk probabilities can be described and quantified by grey system theory, particularly when the system encompasses numerous factors or state variables, as indicated by a vector ${\mathbf{X}}_{{\mathbf{G}}} = (X_{G1} ,\;X_{G2} , \ldots ,\;X_{{G{\text{n}}}} )$ representation. Thus, the system's performance function $g\;({\mathbf{X}}_{{\mathbf{G}}} )$ is determined by these factors, with the system's failure probability defined accordingly. The following concept can be derived similarly, based on the system's critical performance requirements.

1 $$\left\{ \begin{gathered}\ \ [g(X_{G} ) = 0]\; \to \;\lq \lq {\text{Critical}}\;{\text{state}}" \hfill \\ [g(X_{G} ) > 0]\; \to \;\lq \lq{\text{Safe}}\;{\text{state}}" \hfill \\ [g(X_{G} ) < 0]\; \to \;\lq \lq{\text{Failure}}\;{\text{state}}" \hfill \\ \end{gathered} \right.$$

Given the joint probability density function $f_{{_{{X_{G1} }} ,_{{X_{G2} }} ,...,_{{X_{Gn} }} }} (X_{G1} ,X_{G2} ,...,X_{Gn} )$ of the variables $X_{G1} ,X_{G2} , \ldots ,X_{Gn}$, the probability of failure state is expressed as,

2 $$R_{G} = \int\limits_{{g(X_{G} ) < 0}} {f_{{X_{G} }} (X_{G} )} dX_{G}$$

The probability of channel overtopping failure, characterized by grey-stochastic analysis, is

3 $$R_{G} = \left[ {R_{*} ,\;\;R^{*} } \right] = P\left\{ {g({\mathbf{X}}_{{\mathbf{G}}} ) < 0} \right\}$$

More detailed information about the grey systems theory can be found in reference^34^.

#### Instability failure probability model considering stochastic and fuzzy uncertainty

The instability of the channel slope is attributed to the sliding torque $M_{S}$ exceeding the anti-sliding torque $M_{R}$. The failure probability of instability considers fluctuations in the water level $H$ within the channel and the variability of the soil's physical and mechanical properties on the channel slope. Figure 3b depicts the risk probability model for levees' instability failure. Given the complexity of directly solving the risk associated with channel slope instability, discrete numerical integration is commonly utilized. Initially, the section of the load probability density distribution curve $h \le h_{2}$ is divided into $N$ segments, and the subsequent equation is applied for resolution:

4 $$P_{fs} = p(M_{s} > M_{R} ) = \int_{{h_{1} }}^{{h_{2} }} {F_{{M_{S} }} (H) \cdot f_{0} (H){\text{d}}H} = \sum\limits_{i = 1}^{N} {\overline{F}_{{M_{s} }} (h_{i} ) \cdot \Delta F_{0} (h_{i} )}$$

In this equation, $h_{1}$ and $h_{2}$ are the lowest and highest water level values (m) specified when the soil channel slope is unstable;$\Delta F_{0} (h_{i} )$ is the $i$ segment of the water level frequency curve probability;$N$ is the number of segments for calculating the water level frequency curve; $F_{{M_{s} }} (h_{i} )$ is the probability that the sliding torque surpasses the anti-sliding torque at a specified water level $h$; $\overline{F}_{{M_{s} }} (h_{i} )$ is the average probability value of $i$ segment where the sliding torque exceeds the anti-sliding torque.

Assessing the soil's physical and mechanical properties when estimating the probability of channel slope instability presents considerable uncertainty. Thus, a fuzzy failure probability model for channel levee failure is proposed, utilizing fuzzy event probability theory^35^.

5 $$P(\widetilde{{Z_{s} }}) = \int_{U} {\mu_{{\widetilde{{Z_{s} }}}} } (z)f_{s} (z)dz$$

where $\widetilde{{Z_{s} }}$ represents the fuzzy event of channel slope instability, $f_{s} (z) = F_{{M_{S} }} (H) \cdot f_{0} (H)$,$\mu_{{\widetilde{{Z_{s} }}}} (z)$ is the membership function of levee breakdown.

To address the fuzzy uncertainty in instability risk, an ascending semi-trapezoidal distribution is selected, with the membership function defined as follows.

6 $$\mu_{A} (z) = \left\{ \begin{gathered} 0,\quad \quad \quad \quad \quad \quad \quad \quad z < a \hfill \\ (z - a)/(b - a),\quad \;\;a \le z \le b \hfill \\ 1,\quad \quad \quad \quad \quad \quad \quad \quad z > b \hfill \\ \end{gathered} \right.$$

The membership function of this fuzzy failure state $\widetilde{A}$ is expressed as the distribution density function when the state variables $Z$ follow a normal distribution, illustrated in Eq. (7).

7 $$f(z) = \frac{1}{{\delta \sqrt {2\pi } }}\exp ( - (z - \mu )^{2} /2\delta^{2} )$$

The fuzzy failure probability is subsequently calculated as follows.

8 $$\begin{gathered} P_{r} = P(A) = f(z) = \int_{ - \infty }^{\infty } {\mu_{A} (z)f(z)dz} \hfill \\ \quad \; = \int_{\infty }^{b} {\frac{1}{{\delta \sqrt {2\pi } }}\exp ( - (z - \mu )^{2} /2\delta^{2} )dz} + \int_{a}^{b} {\frac{z - a}{{b - a}} \cdot \frac{1}{{\delta \sqrt {2\pi } }}\exp ( - (z - \mu )^{2} /2\delta^{2} )dz} \hfill \\ \end{gathered}$$

#### Seepage failure probability model under stochastic uncertainty

Seepage theory elucidates that seepage deformation (such as pipe surge or flow soil) occurs when the seepage gradient $J$ surpasses the soil's critical gradient $j_{k}$. Moreover, this gradient is influenced by the channel's water level, underscoring the necessity of a comprehensive consideration of hydrological risk.

The probability model for levee seepage failure is depicted in Fig. 3c. This model considers the impact on the structure to commence at a certain water level, reaching its maximum when the water level aligns with the levee's height.

9 $$P_{fi} { = }\int_{{H_{1} }}^{{H_{2} }} {F_{J} (H)f(H)\;{\text{d}}H}$$

where $f(H)$ is the probability density function of water level and $F_{J} (H) = \int_{{j_{k} }}^{\infty } {f(J/H){\text{d}}J}$.

As the most widely used probabilistic methods, mathematical statistical methods, such as the direct integration method, Monte Carlo simulation (MCS) method, and structural reliability method etc., are commonly used to solve risk-rate models with stochastic uncertainty^11^. MCS method is one of the common methods for predicting and estimating failure probability, and it does not need to consider the complex mechanism of influence between random variables. The MCS method's main advantage is its high accuracy, especially for nonlinear, differentially distributed, correlated systems. As a result, the MCS method was used in this paper to solve the failure probability model.

### Integrated failure probability based on the Copula function method

The occurrence of a levee breach in open channels within long-distance water transfer projects is the culmination of a complex interplay among multiple failure modes. This complexity is further amplified by the fact that individual risk factors are not entirely independent; instead, they exhibit mutual penetration and correlation. The Copula method provides a robust framework for analyzing the interrelationships between these failure modes.

Copula theory was first proposed by Sklar, known as Sklar's theorem^24^. Sklar argued that any multivariate joint distribution can be written in terms of univariate marginal distribution functions and a copula which describes the dependence structure between the two variables.

Taking the binary Copula function as an example, let $H\left( {X,Y} \right)$ be a two-dimensional distribution function with marginal distribution functions $F\left( X \right)$ and $G\left( Y \right)$. Then there exists a copula $C$ such that $H\left( {X,Y} \right) = C\left( {F(X),G(Y)} \right)$. Conversely, for any distribution functions $F$,$G$ and any copula $C$, the function $H\left( {X,Y} \right)$ defined above is a two-dimensional distribution function with marginals $F\left( X \right)$ and $G\left( Y \right)$. Furthermore, if $F\left( X \right)$ and $G\left( Y \right)$ are continuous,$C$ is unique.

The binary distribution function and its parameter ranges of Gaussian Copula function are summarized in Table 2.

**Table 2:** Gaussian Copula function.

Copula function	Binary distribution function $C_{\theta } \left( {u,v} \right)$	Parameter ranges	Kendell’s rank correlation coefficient
Gaussian Copula	$\phi_{\rho } \left( {\phi^{ - 1} \left( u \right),\phi^{ - 1} \left( v \right)} \right)$	$\rho \in \left( { - 1,1} \right)$	$\tau = \frac{2\arcsin \left( \rho \right)}{\pi }$

Modes of levee failure can be viewed as a series relationship because no failure modes are permitted during channel operation. The boundary method, a prevalent approximation for the series model, assesses the correlation of failure modes under two extreme conditions: complete correlation and complete independence. This method posits that the correlation among potential risk factors for channel levees lies between these extremes. Therefore, based on De Morgan's Law^36^, when $P_{{E_{j} }} < 1$ the general bound for $P_{t}$

10 $$\mathop {\max }\limits_{1 \le j \le m} (P_{{E_{j} }} ) \le P_{t} \le \sum\limits_{j = 1}^{m} {P_{{E_{j} }} }$$

In practical calculations of the integrated failure probability of channel levees, it is advisable to consider the upper limit of Eq. (10). In this paper, The Copula function was used to estimate the system's failure probability because of its excellent performance in describing the correlation of multivariate variables, as well as its convenience in constructing the joint probability distribution function.

The general correlation influences projects failure probability by influencing the joint failure probability of two failure modes occurring concurrently. The performance function corresponding to each failure mode of the binary series model in the channel levee structure is assumed to be

11 $$g_{i} (X) = g_{i} (X_{1} ,X_{2} ,...X_{n} ),i = 1,2,3....n$$

Subsequently, the probability of simultaneous occurrence of the two failure modes in the binary series model is derived using the probability integral transformation, as illustrated below.

12 $$\begin{gathered} P[g_{1} (X) \le 0,g_{2} (X) \le 0] = P\{ F_{{g_{1} }} [g_{1} (X)] \le F_{{g_{1} }} (0),F_{{g_{2} }} [g_{2} (X)] \le F_{{g_{2} }} (0)\} \hfill \\ \quad \quad \quad \quad \quad \quad \quad \quad \quad \quad \quad = P[U_{1} \le F_{{g_{1} }} (0),U_{2} \le F_{{g_{2} }} (0)] = C[F_{{g_{1} }} (0),F_{{g_{2} }} (0)] = C(P_{{fg_{1} }} ,P_{{fg_{2} }} ) \hfill \\ \end{gathered}$$

**Figure 4:**
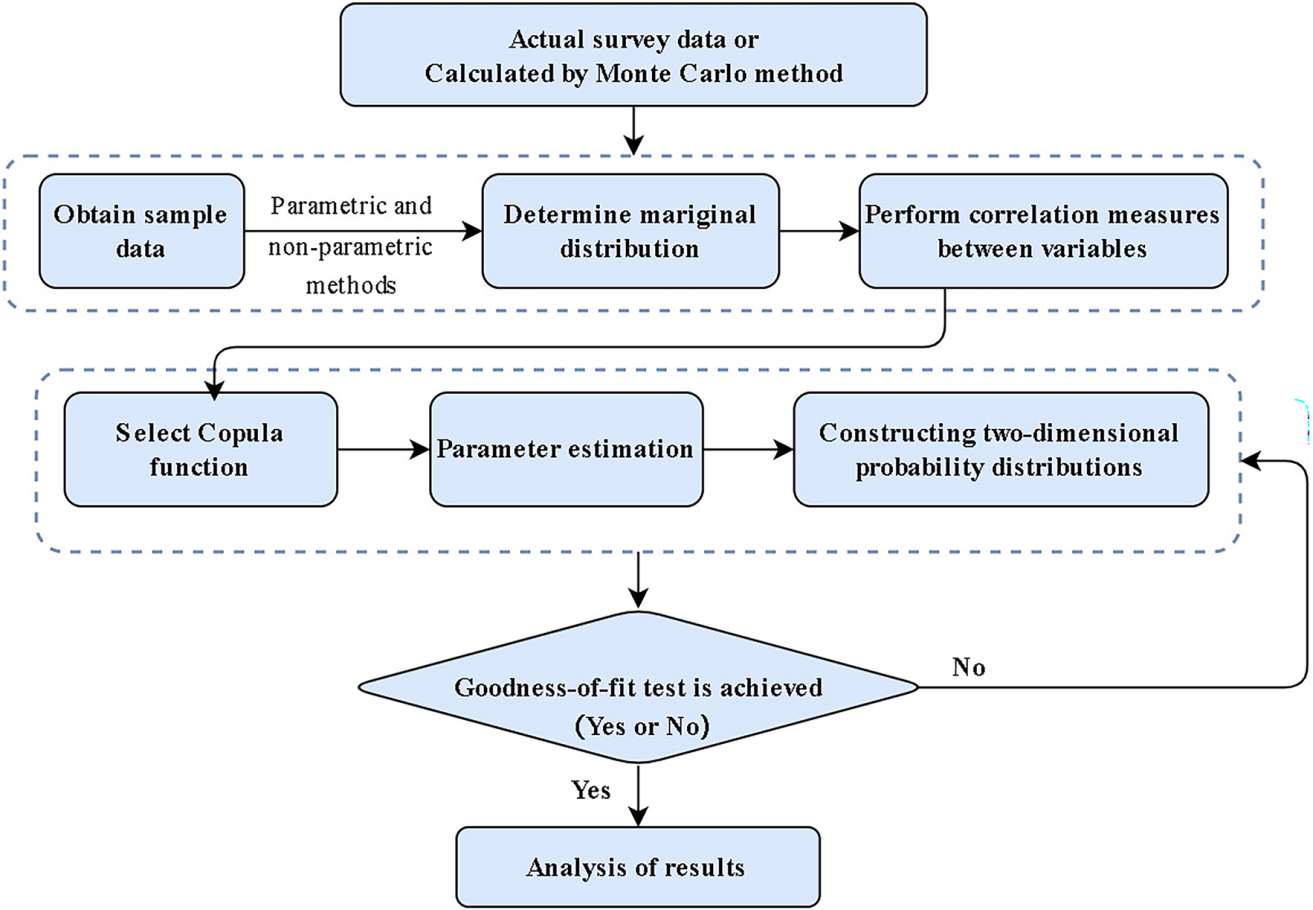
Establishment of a Copula Model.

According to the above, $F_{g} (0,0) = C[F_{{g_{1} }} (0),F_{{g_{2} }} (0)]$ complies with Sklar's theorem. As a result, the failure probability of the binary series model can be calculated.

13 $$\begin{gathered} P_{f} = P[g_{1} (X) \le 0 \cup g_{2} (X) \le 0] \hfill \\ \quad \; = P[g_{1} (X) \le 0) + P(g_{2} (X) \le 0] - P[g_{1} (X) \le 0,g_{2} (X) \le 0] \hfill \\ \quad \; = P_{{fg_{1} }} + P_{{fg_{2} }} - C(P_{{fg_{1} }} ,P_{{fg_{2} }} ) \hfill \\ \end{gathered}$$

where $P_{{fg_{1} }} ,P_{{fg_{2} }}$ are the failure probabilities of the binary series model's two failure modes in the channel levee structure, respectively; $C$ is the binary Copula function. The establishment process of Copula Model is depicted in Fig. 4.

## Results and discussion

**Figure 5:**
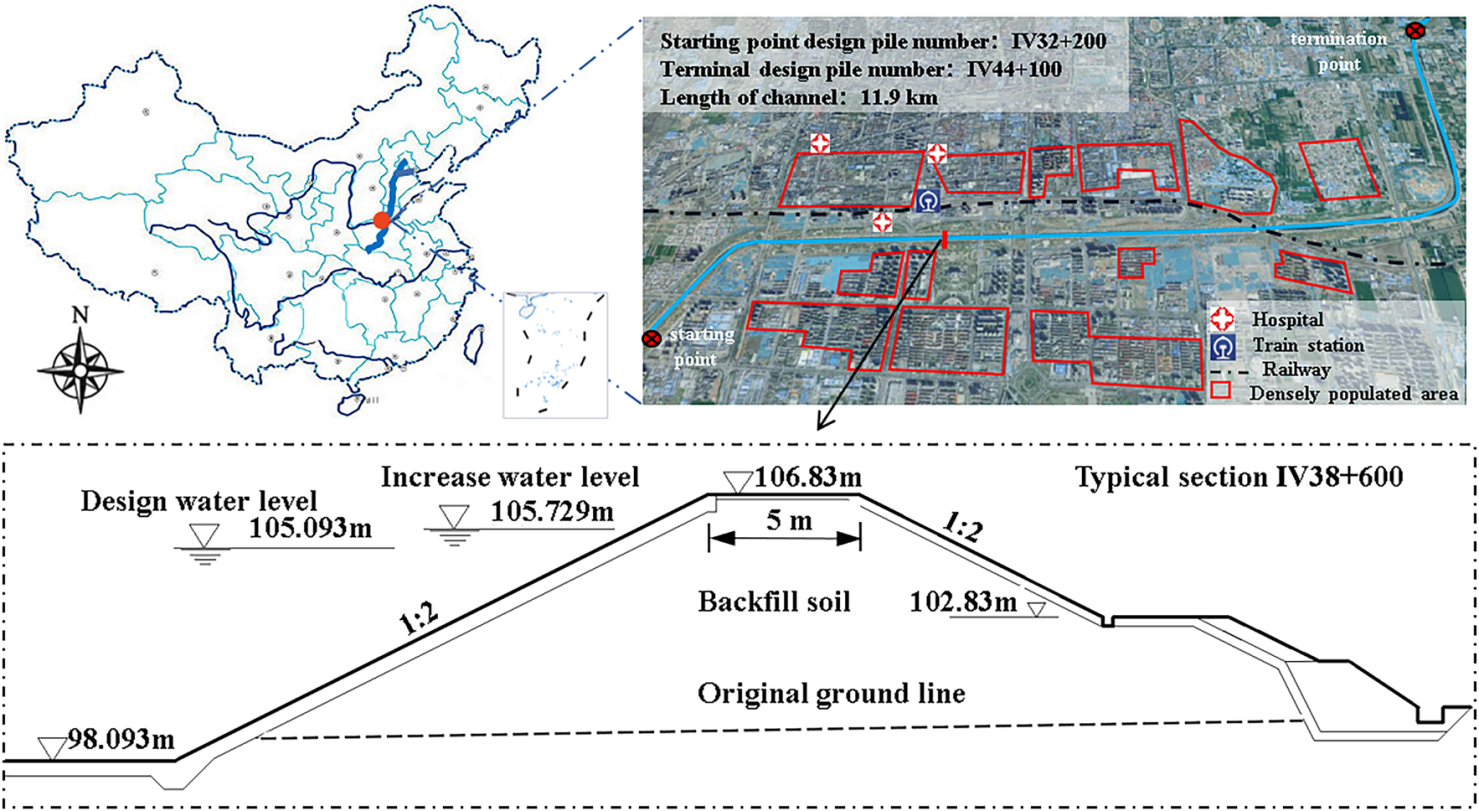
Location of the open channel project and geometry of typical section (IV38 + 600).

A long-distance water transfer project main trunk channel starts from *A* province, the channel line passes through *A, B* and multiple provinces and cities. Notably, the channel section within city J is characterized by high fill, constituting a crucial segment of the project's main canal. This section, spanning from design station IV32 + 200 to IV44 + 100 in city J, lies above a densely populated area. The geographical elevation of this section gradually decreases from northwest to southeast, heightening the risk of levee breaches under atypical operational conditions. Such breaches could release channel water, posing significant threats to the lives and properties of residents along its course. In this study, a typical open channel section in the above area was selected for the application of the probabilistic and non-probabilistic risk analysis model mentioned above. Typical Section IV38 + 600 is a high fill cross-section in city J with a levee crest height of 8.743 m, surrounded by critical infrastructure including train stations, hospitals, and residential areas, thereby indicating a high population density. The engineering location and the geometric profile of a typical cross-section of the study area is illustrated in Fig. 5.

### Estimation of failure probability under multiple uncertainties

#### Grey-stochastic probability of hydrological failure

Based on the water level design standards outlined in Table 3, it can be obtained that the 100-year design water level is 7.533 and the 300-year calibration water level is 8.176 m. When $f(h)$ was used to represent the probability density function of inverse Gaussian distribution, and the $f(7.533) = 0.01$ and $f(8.176) = 0.0033$ can be obtained.

**Table 3:** Channel water level design criteria.

	Recurrence period (year)	Flow (m^3^/s)	Elevation of water level (m)
Design	100–50	265	105.626–104.624
Calibration	300–200	320	106.269–105.255

To solve the system of binary nonlinear equations derived from the aforementioned setup, the Newton iteration method was applied, yielding the $\mu$ and $\lambda$ parameters of the inverse Gaussian distribution. The backwater level frequency curve of the channel was obtained as

14 $$f(h) = \sqrt {\frac{\lambda }{{2\pi h^{3} }}} e^{{ - \frac{{\lambda (h - \mu )^{2} }}{{2\mu^{2} h}}}}$$

The frequency curve of backwater level conforms to the distribution characteristics of backwater level. The probability of overflow and levee breach in the middle canal section of the study area had met the requirements and can be ignored by combining equation in Fig. 3a.

The measured data show that the elevation of the levee top follows a normal distribution. The parameters used to calculate wave run-up and wind backwater height are presented in Table 4. According to measured data and relevant standards, the Putian wind-wave formula^37^ was introduced to calculate the mean value of wave run-up height $\mu_{h}$ and the wind backwater height $e$, and the results are shown in Table 5. The risk variables such as levee top elevation, wave run-up and wind backwater height are subject to grey uncertainty, classifying the channel levee system as a grey uncertainty system. The Dempster-Shafer method^38^ was subsequently applied to transform each risk characteristic parameter calculated for the levee, as shown in Table 5, into grey interval data, as illustrated in Table 6.

**Table 4:** Calculation parameters of wave run-up and wind backwater height.

Parameters	$v$(m/s)	$F$(m)	$h_{a}$(m)	$m$	$K_{\Delta }$	$K_{v}$	$g$(m/s^2^)	$K$	$\alpha$
Values	2.9	100	7.0	2	0.85	1.1	9.81	3.6×10–6	0

**Table 5:** Channel levee risk characterization parameters.

	Levee top elevation (m)	Wave run-up (m)	Wind backwater height (m)
Range of values	8.593–8.893	0.34–0.55	0.00021–0.00023
Average value	8.743	0.48	0.00022
Mean square error	0.05	0.33	0.00001

**Table 6:** Upper and lower grey expectation values of risk characteristics parameters.

Expected Value	Levee top elevation (m)	Wave run-up (m)	Wind backwater height (m)
$E_{G}^{ * }$	9.18	0.504	0.0.00023
$E_{{G^{*} }}$	8.31	0.456	0.0.00021

The methodology for calculating the grey-stochastic risk probability of hydrological failure, as detailed in Section “Hydrological failure probability model considering grey-stochastic uncertainty”, was employed. The failure impact factors were represented by vectors $X_{G} = (X_{1G} ,\;X_{2G} ,\;X_{3G} )$, with an assumption of mutual independence among all factors. The performance function $g(X_{G} ) = g(X_{1G} ,\;X_{2G} ,\;X_{3G} )$ of the channel's safety state can be built.

The minimum value of the failure probability is $2.21 \times 10^{ - 8}$, obtained from the $X_{G*}$ formed by $(X_{1G*} ,\;X_{2G}^{ * } ,\;X_{3G}^{ * } )$, and the maximum value of the failure probability is $1.27 \times 10^{ - 7}$ obtained from the $X_{G}^{ * }$ formed by $(X_{1G}^{ * } ,\;X_{2G*} ,\;X_{3G*} )$. Therefore, the grey-stochastic failure probability $R_{G}$ of hydrological failure in the study channel segment is $[2.21 \times 10^{ - 8} ,\;1.27 \times 10^{ - 7} ]$. When grey uncertainty is not taken into account, the hydrological failure probability is $7.18309 \times 10^{ - 8}$, within the interval range of the grey-stochastic failure probability. This approach, which incorporates both random and grey uncertainties, yields an interval value for the hydrological failure probability, offering a more comprehensive and accurate assessment of the uncertainty associated with failure modes.

#### Fuzzy-stochastic probability of instability failure

The stability of levee slopes is significantly influenced by the variability in the soil's physical–mechanical properties. The properties of levee materials in the examined channel section are detailed in Table 7. Generally, the variability of the channel levee's geometric parameters and the unit weight of its materials is very small and is treated here using constant values. The statistical characteristics of the soil shear index of the channel levee are shown in Table 8.

**Table 7:** Physical and mechanical characteristics of study area soil.

Soil classification	Cohesive strength $c$ (kPa)	Angle of friction $\varphi$ (°)	Dry density $\gamma$ (kN/m^3^)	Saturation capacity $\gamma_{w}$ (kN/m^3^)
Heavy silt loam	36.0	19.5	1.78	2.03
Silty clay	32.0	22.6	1.68	1.99

**Table 8:** Statistical characteristics of shear resistance index of study area soil.

Soil	Parameters	Mean value	Standard deviation	Distribution pattern
Heavy silt loam	Cohesive strength $c$ (kPa)	40.76471	9.680359	Normal distribution
	Angle of friction $\varphi$(°)	24.17059	3.514301	Normal distribution

**Figure 6:**
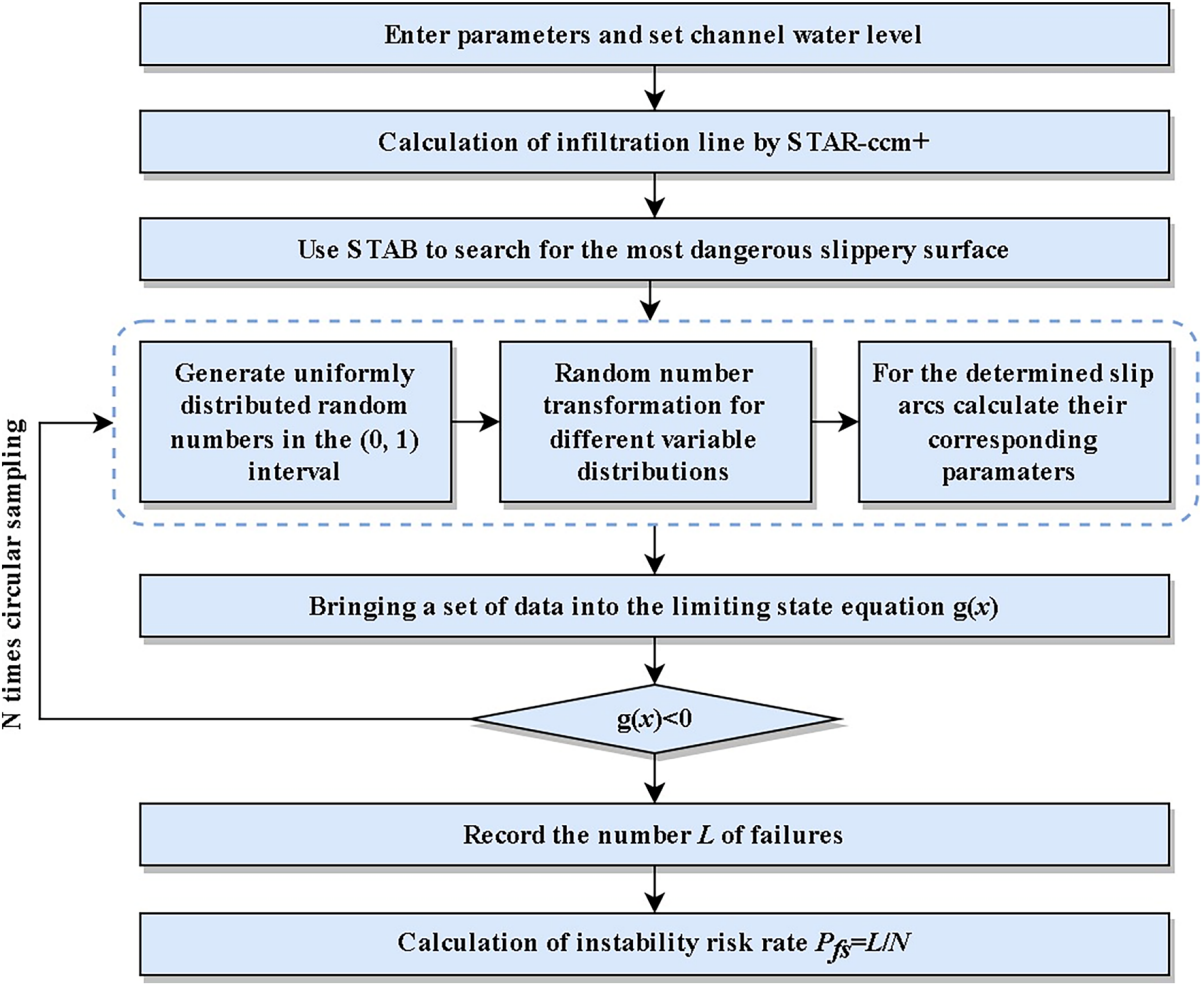
Simplified Bishop's law calculation flow chart.

The simplified Bishop method calculated the risk of channel slope instability, described by the limit state equation as follows.

15 $$g(x) = \left\{ {\sum {[C_{i} b_{i} + (W_{i} - U_{i} b_{i} )\tan \varphi_{i} ]} \sec \theta_{i} /(1 + \tan \varphi_{i} \tan \theta_{i} )} \right\} - \sum {W_{i} \sin \theta_{i} }$$

where $C_{i}$(kPa),$\varphi_{i}$(°) is the shear strength index of soil; $U_{i}$ is pore pressure (kPa); $W_{i}$ is the self-gravity of the soil strip (kN); $b_{i}$ is the width of soil strip (m); $\theta_{i}$ is the Angle between the tangent line of the midpoint at the bottom of the soil strip and the horizontal line (°). The procedure for solving risk of sliding instability is shown in Fig. 6.

**Figure 7:**
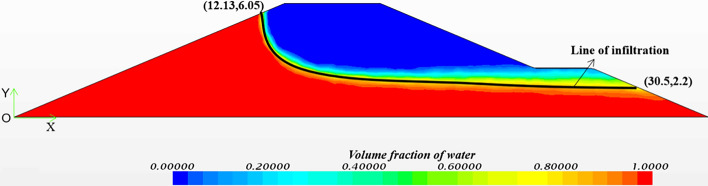
Channel levee seepage infiltration line.

The infiltration line of channel levee seepage flow under water level conditions at all levels can be obtained by numerical simulation of channel levee seepage flow, with calculations performed at a channel water level of 8.737 m, as shown in Fig. 7.

**Figure 8:**
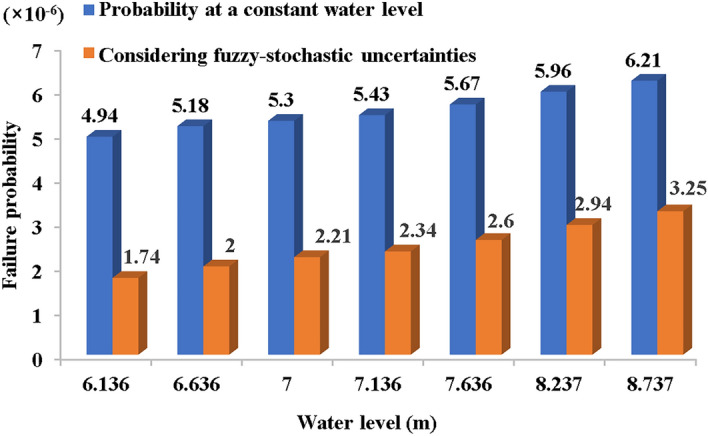
Failure probability of instability under different water level.

Using numerical simulations to obtain the levee's infiltration line under different water levels, STAB software analyzed the search for the most critical sliding surface. The safety coefficient of the circular arc sliding surface was solved by Bishop method and obtain the location of the sliding surface with the lowest safety coefficient of Bishop method. The MCS method evaluated the risk of sliding instability, utilizing the most hazardous sliding arc identified by STAB software, as illustrated in Fig. 6. The stochastic-fuzzy risk of instability of the channel levee slope under each water level condition is shown in Fig. 8.

The fuzzy uncertainty in the risk of channel levee instability failure was addressed using the ascending semi-trapezoidal distribution, and the membership degree of limit state $$g(x)$$ was obtained at each water level in the channel. As observed in Fig. 8, the fuzzy-stochastic failure probability is minor compared to scenarios excluding fuzzy-stochastic uncertainty consideration. Both failure probabilities escalate with water levels, with their difference diminishing at higher levels. The reason for this phenomenon is that the higher the water level is, the greater the membership degree of $$g(x) < 0$$ is, which reveals the randomness and ambiguity of $$C_{i}$$$$\phi_{i}$$.

In addition, the failure probability analysis across different water levels demonstrates a gradual increase in risk with rising water levels, albeit at a slow rate, indicating the channel slope instability risk's insensitivity to water level variations. With a 2.2-m increase in water level, the failure probability doubles. At a channel water level of 8.737 m, the probability of instability failure is merely 0.000325%. Without considering fuzzy-stochastic uncertainties, the failure probability is $3.87 \times 10^{ - 5}$, while the fuzzy-stochastic failure probability is $P_{fs} = 1.71 \times 10^{ - 5}$, underscoring the value of incorporating more detailed information into the analysis.

#### Stochastic probability of seepage failure

Based on the numerical simulation of seepage field, the anti-seepage measures failure took into consideration. The elevation of the overflow point under each water level condition was obtained, and the seepage slope under various water levels was calculated based on the numerical simulation of the seepage field. The outcomes of these calculations are summarized in Table 9.

**Table 9:** Calculation results of seepage point elevation and hydraulic gradient.

Water level (m)	5.636	6.136	6.636	7.000	7.136	7.636	8.237	8.737
Elevation of seepage point (m)	1.39	1.59	1.70	1.79	1.87	2.14	2.31	2.46
Maximum seepage gradient	0.29	0.38	0.44	0.40	0.37	0.46	0.50	0.55

Took the actual section size weakening and calculation simplification, calculation errors and other factors into considerations, $J$ is uncertain. Assuming that $J$ follows triangular distribution and the calculated $J$ is reliable, the maximum value was estimated to be $1.2J$, and the minimum value was estimated to be $0.4J$.

**Figure 9:**
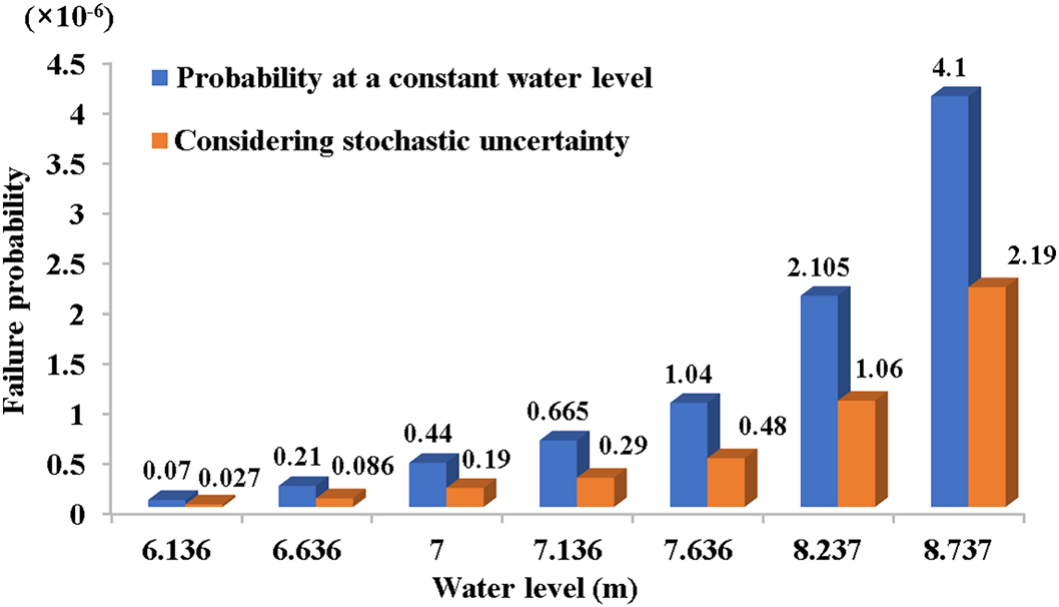
Failure probability of seepage under different water level.

A performance function for the seepage failure mode of the channel was developed, allowing for the calculation of the probability that the seepage gradient exceeds the critical gradient at specific water levels using the MCS method. The failure probability at each water level is shown in Fig. 9.

It is demonstrated in Fig. 9 that the seepage failure probability of this channel levee section is sensitive to the change of channel water level. Specifically, at a water level of 8.737 m, the seepage failure probability stands at 0.00219, whereas at 6.6136 m, it reduces to merely 0.0000266. An increase of 2.2 m in the water level results in an 82-fold increase in failure probability, with a more rapid escalation observed at higher water levels. The seepage failure probability, excluding stochastic uncertainty, is calculated as 0.00863. Add the failure probability at each water level to obtain the permeability failure probability value $P_{fi} = 0.004333$. The result of failing to account for uncertainty is overestimated. Among them, the failure probability value at a water level of 8.737 m, which accounts for over half of the probability value. It is shown that if the levee was under long-term high water level operation, it will be more prone to levee breach caused by seepage damage.

**Figure 10:**
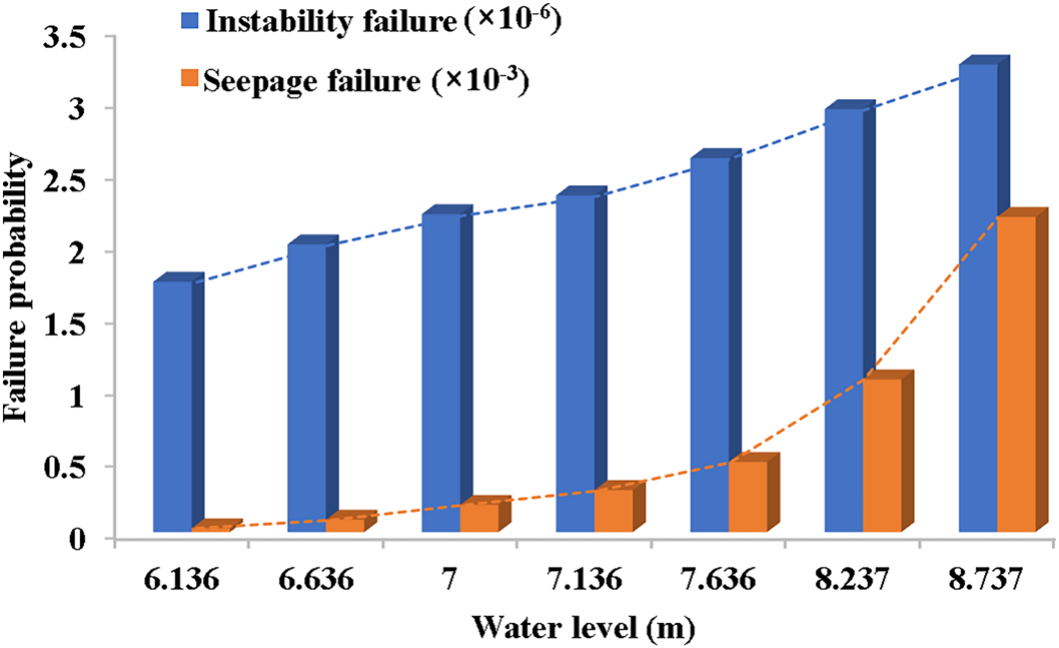
Structural failure probability at different water levels.

As shown in Fig. 10, two structural failure probabilities are analyzed at different water levels, considering uncertainty. It is evident from Fig. 10 that failure probability demonstrates low sensitivity to water level changes at lower levels but significantly increases at higher levels. Notably, seepage failure exhibits greater sensitivity to water level alterations compared to instability failure.

In accordance with Eq. (10) and the aforementioned analysis, the integrated failure probability of levee breaching can be obtained as 0.435%. The integrated failure probability mainly comes from the contribution of seepage failure, which is sensitive to water level changes. And this sensitivity increases as the water level gets higher. Although hydrological and instability failures contribute less to the overall failure probability, their influence grows with rising water levels. Therefore, closely monitoring water level changes, especially during the flood season, can substantially mitigate the risk of channel levee failure.

### Integrated failure probability considering correlated failure modes in levee breach event

It is recognized that there exist complicated correlations among different failure modes due to the shared uncertain variables in the context of levee breach^39^. For instance, both seepage failure and instability failure are related to soil strength parameters. Furthermore, according to the obtained failure probability in a certain failure mode of channel levee breach, seepage and instability failures contribute more significantly to the integrated failure probability than hydrological failure. Therefore, leveraging the Copula function model introduced in Section “Integrated failure probability based on the Copula function method”, the relationship between seepage and instability failures was modeled as a two-component series, with correlation analysis conducted using the joint probability density function.

**Figure 11:**
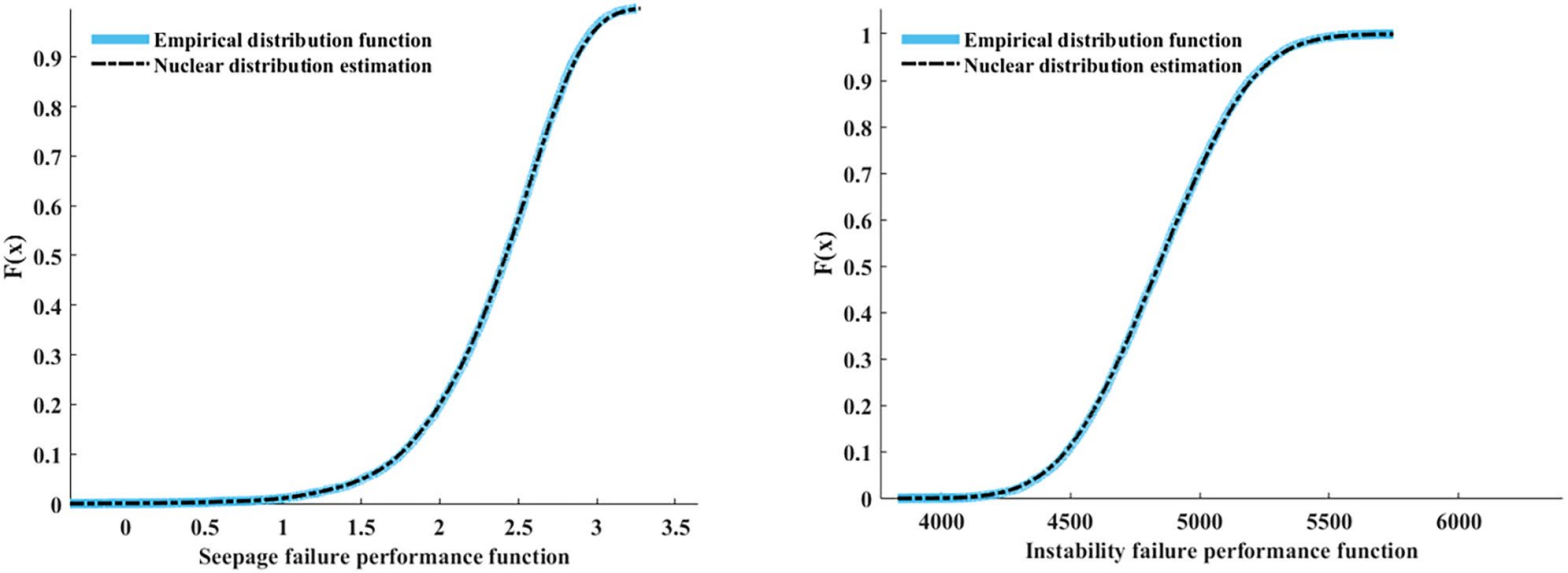
The empirical function and the kernel distribution estimation map.

**Figure 12:**
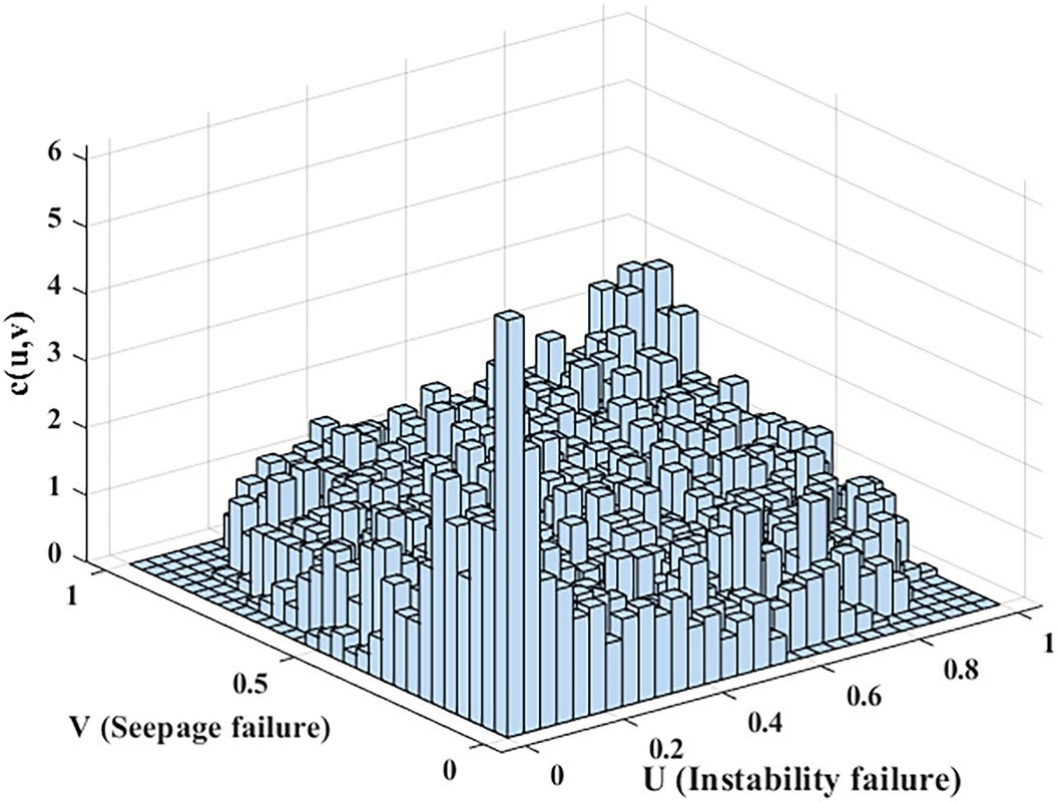
Frequency histogram of seepage and instability failure.

The MATLAB was used in this section. Utilizing performance function sample data for the two failure modes obtained from previous calculations, the performance functions for instability and seepage failures were represented as $X {\text{and }}Y$. To identify an appropriate copula function, the non-parametric kernel density estimation method^40^ was employed to estimate the marginal probability density distributions for both failure modes (Fig. 11), followed by generating a binary frequency histogram (Fig. 12).

As shown in Fig. 12, the binary frequency histogram has a basic symmetric shape and tail, indicating that the joint probability density function will also have a basic symmetric shape and tail. Therefore, the binary normal (Gaussian) Copula function suitable for describing the correlation structure between seepage and instability failures.

**Figure 13:**
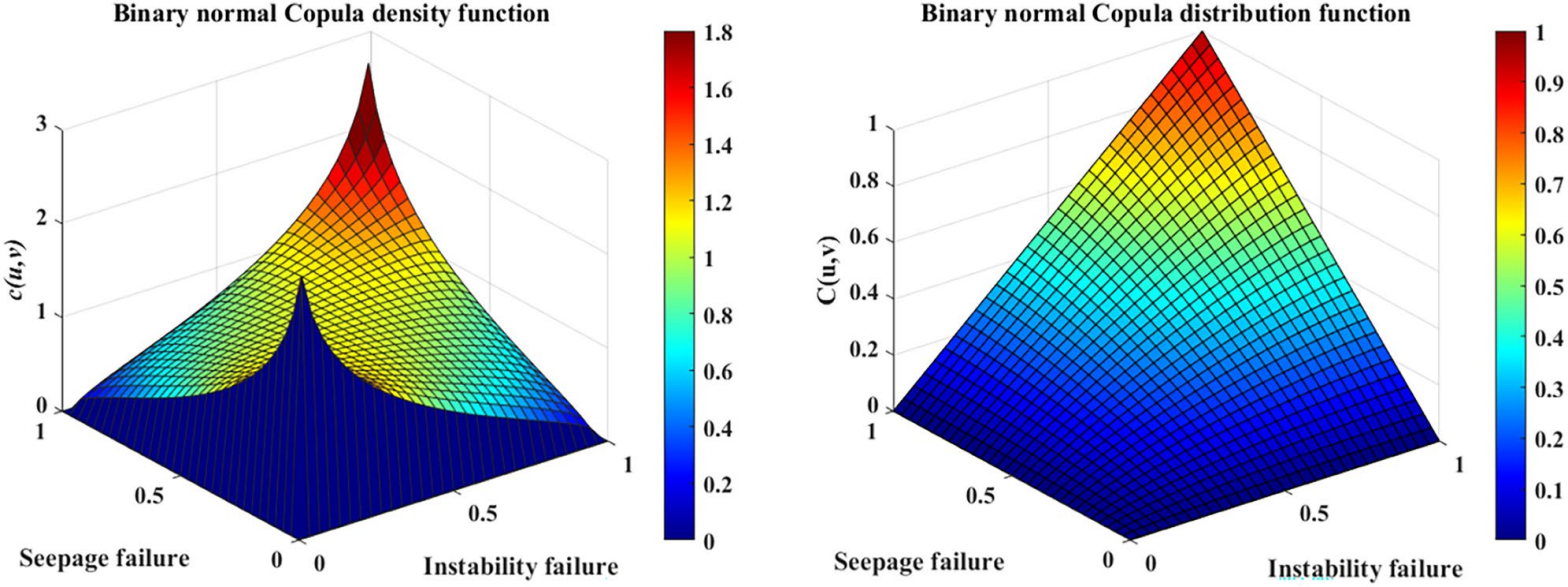
Gaussian Copula density function and distribution function ($\rho = 0.3821$).

In MATLAB, the copulafit function was used to estimate the correlation parameters in the Copula function. The estimated value of the correlation parameter in the function was obtained, $\rho = 0.3821$. The Copula function of the two-component series model was obtained by substituting the calculated parameters into the Eq. (16).

16 $$C(u,v) = \int_{ - \infty }^{{\varphi^{ - 1} (u)}} {\int_{ - \infty }^{{\varphi^{ - 1} (v)}} {\frac{1}{{2\pi \sqrt {1 - \rho^{2} } }}} } \exp \left[ { - \frac{{x^{2} - 2\rho xy + y^{2} }}{{2(1 - \rho^{2} )}}} \right]{\text{d}}x{\text{d}}y$$

where $\varphi^{ - 1} ({)}$ is the inverse function of the one-dimensional standard normal distribution function, $\rho$ represents the correlation parameters. The binary normal Copula density function and distribution function were calculated and as shown in Fig. 13.

The Kendall’s and Spearman’s rank correlation coefficients for the Gaussian Copula function were calculated. Meanwhile the Pearson correlation coefficient, Kendall’s and Spearman’s rank correlation coefficient of the original data were calculated, and the results are shown in Table 10.

**Table 10:** Comparison of copula function parameters.

	Correlation parameters $\rho$	Kendall’s rank correlation coefficient	Spearman’s rank correlation coefficient
Gaussian Copula	0.3821	0.2496	0.3671
Sample Data	0.3807	0.2373	0.3512
Relative error	0.0014	0.0123	0.0109

It can be seen that the values of the Gaussian Copula function's parameters have small error values when compared to the parameters of sample data. Thus, the Gaussian Copula function better reflects the relationship between instability and seepage failure of channel levee.

The magnitude of the squared Euclidean distance between the binary normal Copula function and the empirical Copula function is calculated using the MATLAB distribution to assess the model's merit. The squared Euclidean distance $d^{2}$ reflects the fitting accuracy of the original sample data by the binary Copula function, and the smaller the value is, the better the fitting result will be^41^. The computational analysis yielded a numerical result of $d^{2} = 0.2762$, indicating that the chosen binary normal Copula function model can better fit the correlation structure of the original data.

The Patton Copula toolbox was applied to calculate $C(P_{{fg_{1} }} ,P_{{fg_{2} }} ) = 1.196 \times 10^{ - 5}$. Based on the Copula function, the correlation between instability failure and seepage failure was analyzed and the integrated failure probability $4.3351 \times 10^{ - 3}$ was obtained according to Eq. (13).

In view of the correlation calculation of failure modes for long-distance water transfer projects, scholars adopted the approximate reconstruction method of system safety margin equation to find the correlation between failure modes of water transfer project systems^42^. First, a linearized model of the safety margin equation ${\text{G}}_{11} ,G_{12} ,G_{13}$ corresponding to the three failure modes was obtained. Then, the equivalent failure boundary ${\text{G}}_{1e}^{(1)}$ of ${\text{G}}_{11}$ and $G_{12}$ is obtained through equivalence. Finally, by equating ${\text{G}}_{1e}^{(1)}$ and $G_{13}$,the correlation between the three failure modes was obtained.

In order to verify the rationality of the correlation results presented in this paper, we equivalent the safety margin equations linearized models $G_{12}$ and $G_{13}$ of the safety margin equations of the instability failure and the seepage failure using the equivalence method in the literature. The correlation between instability and seepage failure of this project was obtained as $\rho_{{{\text{G}}_{12} G_{13} }} = 0.3791$. The correlation coefficient calculated based on Copula function in this paper is close to the correlation coefficient of the literature. The rationality of this method is illustrated.

The integrated failure probability, determined via the Copula function, presents a more accurate assessment than the boundary method, enhancing decision-making for the project's long-term stability. Considering the project's significance and the emphasis on minimizing hydrological and instability failures during design and construction, the seepage failure probability emerges as a critical concern in long-term operations. In the long-term operation of the channel, the failure probability of seepage is relatively large. Therefore, the failure probability of seepage is much larger than the probability of instability failure and hydrologic failure. As a result, the integrated failure probability considering correlation is small. Assuming that the probability of seepage failure and instability failure values are both large, the calculated integrated failure probability considering the failure modes correlation is more obvious and has more decision reference value.

## Conclusions

The risk assessment of an open channel is critical to the safe operation of a long-distance water transfer project. And its operation system is complicated, and there are numerous uncertainties in the process. Presently, risk analyses have primarily focused on stochastic uncertainties within hydraulic engineering systems, often overlooking the integral aspects of fuzzy and grey uncertainties involved in the project. The grey, stochastic and fuzzy uncertainties are three inseparable uncertainty factors that influence channel levee safety and exist objectively in high fill levee construction. In addition, the failure mode correlation analysis is an important component of the failure probability of channel levee in long-distance water transfer projects. To that end, it is imperative to account for grey, stochastic, and fuzzy uncertainties, as well as the interrelations among failure modes, in high fill levee analyses. This study has quantified uncertainty through a combination of probabilistic and non-probabilistic methods, establishing a failure probability model for long-distance water transfer project levees that considers multiple uncertainties. The correlation among failure modes was explored, enhancing the traditional risk analysis framework beyond the limitations of linear correlation and complete independence. From this research, several key conclusions have been drawn:

- The methodology employed in this research considers not only engineering random uncertainty, but also fuzzy uncertainty and grey uncertainty, allowing the risks in the engineering operation to be considered comprehensively. The result of ignoring uncertainty is overestimated, compared with risk analysis considering multiple uncertainties, because of the randomness and fuzziness of some parameters and the greyness of information.
- The study reveals that the failure modes of channel levees are notably sensitive to changes in water level, suggesting that active monitoring and management of water levels during peak flood seasons can significantly mitigate the risk of levee breaches.
- Based on the Copula function, the correlation between instability failure and seepage failure was analyzed and the integrated failure probability $4.3351 \times 10^{ - 3}$ was obtained. The relationship between instability and seepage failure of channel levee are better reflected by Copula function. The traditional approach, which fails to consider the correlation between failure modes, often overestimates the risk associated with levee breaches, particularly when the probabilities of seepage and instability failures are substantial and closely aligned. The integrated failure probability derived from this analysis is vital for the safety monitoring of long-distance water transfer projects.

The method proposed in this study makes up for the failure to consider the greyness, randomness and fuzziness in the risk analysis of the high fill levee. Due to the similarity of structural engineering analysis principles, the analysis method proposed is not limited to the failure probability analysis of high fill levee, but can also be used for the other structural engineering projects. However, the probability distribution of stochastic variables is determined by engineering experience because of insufficient data and there is no uniform standard for the selection of fuzzy failure criteria. In the correlation analysis, only the correlation between instability and seepage failure was calculated due to the high probability of landslide and osmotic instability. Further study is devoted to developing simplified methodology considering the influence of time, and conducting a more in-depth sensitivity analysis, providing more scientific and reasonable decision information for long-term stable operation of long-distance water transfer project.

## Data Availability

All associated data have been presented in the manuscript which are available from the corresponding author on reasonable request.
